# Targeting the dual miRNA/BMP2 network: LncRNA H19-mediated temozolomide resistance unveils novel therapeutic strategies in glioblastoma

**DOI:** 10.3389/fonc.2025.1577221

**Published:** 2025-04-14

**Authors:** Qiudan Chen, Shihao Zhuang, Shuying Chen, Biying Wu, Qingyu Zhou, Weifeng Wang

**Affiliations:** ^1^ Department of Clinical Laboratory, Central Laboratory, Jing’an District Central Hospital of Shanghai, Fudan University, Shanghai, China; ^2^ Department of Pediatrics, Fujian Children’s Hospital, Fuzhou, China; ^3^ Department of Laboratory Medicine, Huashan Hospital, Fudan University, Shanghai, China; ^4^ Department of Clinical Laboratory, Shanghai Fifth People’s Hospital, Fudan University, Shanghai, China; ^5^ Department of Laboratory Medicine, Longhua Hospital, Shanghai University of Traditional Chinese Medicine, Shanghai, China

**Keywords:** glioblastoma, temozolomide, lncRNA H19, bone morphogenetic protein 2, hsa-miR-138-5p, hsa-miR-22-3p

## Abstract

**Background:**

Long noncoding RNA (lncRNA) is known to not only be involved in various biological processes but also to play a crucial role in chemotherapy resistance. The development of resistance in glioblastoma (GBM) poses a significant challenge in clinical settings. Nonetheless, the mechanisms through which lncRNA contributes to acquired resistance to Temozolomide (TMZ) in GBM patients remain unclear.

**Methods:**

We identified 265 upregulated and 396 downregulated lncRNAs associated with chemoresistance in GBM from the GEO database (GSE100736). Subsequently, we assessed the expression levels of lncRNA H19, hsa-miR-138-5p, hsa-miR-22-3p, and BMP2 mRNA through quantitative polymerase chain reaction (qPCR) in GBM cells and TMZ-resistant GBM cells. Cell viability and proliferation were evaluated using CCK-8 and cell colony formation assays, respectively. Apoptosis was determined through flow cytometry analysis. The impact of gene overexpression and knockdown on cell proliferation and apoptosis was examined via cell transfection experiments. Furthermore, we investigated the influence of lncRNA H19 on tumor development using an *in vivo* xenograft tumor model.

**Results:**

The upregulation of lncRNA H19 was observed in TMZ-resistant GBM cell lines and tissues, suggesting its involvement in acquired TMZ resistance. Silencing lncRNA H19 restored TMZ sensitivity in resistant GBM cells *in vitro*. Conversely, overexpression of lncRNA H19 promoted GBM cell proliferation and hindered TMZ-triggered apoptosis, facilitating the acquisition of TMZ resistance. Notably, lncRNA H19 functions as a molecular decoy for hsa-miR-138-5p and hsa-miR-22-3p, and these miRNAs can reverse the acquired TMZ resistance induced by lncRNA H19 in GBM cells. Additionally, BMP2 gene expression is crucial in the lncRNA H19-mediated pathway of acquired TMZ resistance in GBM cells. Knockdown of lncRNA H19 reinstated TMZ sensitivity *in vivo*, whereas BMP2 overexpression reinstated TMZ resistance.

**Conclusion:**

LncRNA H19 enhances TMZ resistance in glioblastoma through competitive RNA targeting of BMP2.

## Introduction

Glioblastoma multiforme (GBM), the most prevalent malignant tumor among central nervous system cancers, is characterized by rapid progression, high aggressiveness, and challenging curative prospects. Despite recent advances in GBM treatment research, many patients still face the grim reality of short survival and poor prognosis (1, 2). The primary treatment approach involves surgical resection followed by adjuvant radiotherapy and chemotherapy (2, 3). However, the emergence of chemoresistance significantly worsens outcomes for GBM patients (4, 5). Therefore, identifying new therapeutic targets is urgently needed to improve chemotherapy effectiveness and prolong patient survival.

Bone morphogenetic proteins (BMPs) are growth factors and morphogens belonging to the transforming growth factor β (TGF-β) superfamily. They regulate bone and cartilage, and participate in diverse biological processes including development and cancer (6). The classical BMP signaling pathway has been extensively studied (7, 8). In this pathway, BMP ligands bind to membrane serine-threonine kinase receptors, forming heterotetrameric complexes that phosphorylate receptor-activated SMAD (9). Activated R-SMAD then complexes with SMAD and translocates into the nucleus, where it cooperates with other transcription factors to regulate gene expression (10, 11). Importantly, expression of BMP1B-type receptors and BMP2 ligands is elevated in glioblastoma multiforme (GBM) compared to low-grade GBM (12, 13). BMP2 expression correlates with GBM tumor malignancy and patient survival, suggesting its potential as a prognostic marker for human GBM (14, 15). Studies have shown that synthetic BMP-2 mimetic peptides induce differentiation of glioblastoma stem cells (GSCs) (16), and enhance GSC sensitivity to TMZ treatment by destabilizing HIF-1 (17, 18). Despite these benefits, GSCs may resist BMP-induced differentiation by upregulating the extracellular antagonist Gremlin 1 (GREM1) (19). However, there is also evidence suggesting that BMP-2 may activate anti-apoptotic signaling (e.g., Bcl-x(L)) through non-classical pathways (e.g., PI3K/AKT or MAPK), thereby enhancing the tolerance of glioma stem cells (GSCs) to microenvironmental stresses (e.g., hypoxia, chemotherapy). Overactivation of the PI3K/AKT pathway is also an important cause of multidrug resistance (20, 21). This could lead to the acquisition of a chemo-resistant phenotype or contribute to the remodeling of the tumor microenvironment via paracrine signaling (22, 23). Further investigation into intracellular mechanisms is thus warranted to optimize BMP-induced differentiation therapy.

Long non-coding RNAs (lncRNAs), exceeding 200bp, play pivotal roles in various biological processes, including resistance to TMZ (24–26). The imprinted lncRNA H19, expressed solely from maternally inherited alleles (27, 28), contributes significantly to breast cancer cell proliferation, metastasis, chemotherapy resistance, and stem cell maintenance (29–31). Specifically, H19 promotes cancer resistance to triamcinolone acetonide through autophagy (32), but its precise role in TMZ resistance development remains unclear. A recent study has shown that the long non-coding RNA (LncRNA) H19 encodes an immune-related protein, H19-IRP, which promotes immunosuppression in glioblastoma multiforme (GBM) by binding to and activating the transcription of the CCL2 and galactaglutinin-9 promoters. This activation, in turn, recruits myeloid-derived suppressor cells (MDSCs) and tumor-associated macrophages (TAMs), leading to T-cell depletion and the establishment of an immunosuppressive GBM tumor microenvironment (TME) (33). Additionally, some researchers have found that the long non-coding RNAs H19 and c-Myc act as upstream inhibitors of miR-29b in GBM cells, resulting in increased expression of the Nerve/glial antigen (NG)2 (34). Moreover, it has been reported that in C6-induced GBM, H19 promotes the switching of autophagy to apoptosis in response to a combination of temozolomide (TMZ) and interferon-γ (IFN-γ) via the miR-29a/autophagy-related protein 9A (ATG9A) pathway (35). Interestingly, autophagy mediated by the PI3K/AKT/mTOR pathway plays a crucial role in tumorigenesis, progression, and drug resistance (21). These findings suggest that H19 plays a critical role in GBM development and resistance.

In our study, we have identified that the upregulation of lncRNA H19 in GBM cells is associated with acquired resistance to TMZ. This resistance mechanism involves lncRNA H19 acting as a sponge for hsa-miR-138-5p and hsa-miR-22-3p, thereby promoting TMZ resistance in GBM cells. This novel molecular mechanism offers promise as a potential biomarker for overcoming TMZ resistance.

## Materials and methods

### Cell culture

The human GBM cell lines U87, LN229 and HEK293T were obtained from the American Typical Culture Collection (ATCC). U87 and LN229 cell lines were cultured in Dulbecco’s modified Eagle medium supplemented with 10% fetal bovine serum, 100 units/mL penicillin, and 100 μg/mL streptomycin (Invitrogen) under standard cell culture conditions of 37°C and 5% CO2 with humidity. To treat the cell lines, a stock solution of temozolomide (TMZ) (Sigma, USA) was prepared by solubilizing it with DMSO (Sigma-Aldrich, St Louis, MO, USA). TMZ was employed to progressively induce *in vitro* TMZ resistance in U87 and LN229 cells, resulting in the generation of TMZ-resistant U87TR and LN229TR cell lines. The induction started with a TMZ concentration of 2.5 μM and concluded at 1 mM, this process lasted about 6 months (36).

### Cell transfection

The oligonucleotides, shRNAs and scrambled shRNA were synthesized by Guangzhou Ige Biotechnology (China). The oligonucleotide sequences were listed in **Supplementary Table 1**. Transfections were performed using Lipofectamine 3000 reagent (cat. no. L3000015; Invitrogen, Carlsbad, CA, USA) according to the manufacturer’s instructions. For lentiviral stable transfection, cells were seeded at 50% confluence in a six-well cell culture plate containing complete growth medium. Knockdown and overexpression efficiency were confirmed by qRT-PCR and/or Western Blot.

### Cell viability assay

To assess cell viability, we used the CCK-8 assay. Initially, the cells were seeded into 96-well plates at a density of 4000 cells per well. Following 48 hours of incubation, 10% CCK-8 was introduced into each well and incubated for a period of 2 hours. The absorbance was measured at 450 nm using a Thermo Varioskan Flash reader (Thermo Fisher Scientific, Waltham, MA, USA).

### RNA extraction and real-time quantitative PCR assay

Total RNA was extracted from cultured cells using TRIzol reagent (Invitrogen, CA, USA). The cDNA was synthesized by reverse transcription of total RNA using the PrimeScript RT kit (Takara, Nanjing, China). Real-time quantitative PCR (qRT-PCR) analysis was performed using the SYBR Green Premix Ex Taq (Takara, Nanjing, China). The levels of lncRNA H19 and mRNA were normalized by Glyceraldehyde 3-phosphate dehydrogenase (GAPDH). Relative RNA expression levels were determined using an ABI 7500 real-time PCR system (Applied Biosystems, Foster City, CA, USA). The primer sequences for lncRNA H19 were as follows: 5’-ACTCAGGAATCGGCTCTGGAA-3’ (forward primer) and 5’-CTGCTGTTCCGATGGTGTCTT-3’ (reverse primer). The relative quantitative values of lncRNA H19 and mRNA were expressed as 2^-ΔΔCT^.

### Western blot analysis

Cells were cultured in 6-well plates with 1 × 10^6^ cells and incubated for 24 hours. After processing, cells were lysed using RIPA buffer (Pierce, Rockford, USA). Protein concentration was determined using the BCA Protein Assay Kit (cat#: P0012, Beyotime, China). Proteins were separated on 12% SDS polyacrylamide gels and transferred to Polyvinylidene fluoride (PVDF) membranes (Merck Millipore, Billerica, USA). Primary antibodies, including α-tubulin (Cell Signaling Technology, Cat#4697S) and BMP2 (Merck, Cat. No. ABN898), were used at a dilution of 1:1000. Protein bands were detected using horseradish peroxidase (HRP)-conjugated antibodies (MultiSciences, GAM0072 and GAR007) and visualized by enhanced chemiluminescence (Merck Millipore, Billerica, USA).

### Enzyme-linked Immunosorbent Assay (ELISA)

ELISA was employed to quantify BMP2 levels in the serum using the BMP-2 ELISA Development Kit (ABTS) purchased from Thermo Fisher Scientific (Catalog # 900-K255K, Bremen, Germany).

### Cell colony formation assay

To perform the cell colony formation assay, 500 cells per well were inoculated into 6-well culture plates and cultured in DMEM containing 10% FBS. After receiving the indicated treatments, the cells were incubated at 37°C and 5% CO_2_ for approximately two weeks. The colonies were then stained and counted using 0.1% crystal violet (Sigma-Aldrich). Three independent experiments were performed for each set of clones.

### Flow cytometry analysis of apoptosis

Apoptosis assays of GBM cells were performed using the apoptosis analysis kit (Roche) after the indicated treatments. Finally, cells were analyzed using flow cytometry (BD FACSCanto II).

### Cell derived xenograft model

For *in vivo* studies, 5-week-old male BALB/C nude mice were purchased from Shanghai Lab Animal Research Center (China) and maintained in a specific pathogen-free (SPF) facility. For each xenograft model, a total of 2 × 10^6^ cells was collected and injected subcutaneously into the right back of nude mice, with 5 mice in each group. Mice were orally administered TMZ (66 mg/kg per day for 5 days) at 2 weeks after surgery. Tumor volume = 0.5 × (length) × (width)^2^ (37). Animals were euthanized if the tumor volume exceeded 2000 mm³; or >20% body weight loss within 72 hours or severe lethargy/inability to access food/water. Euthanasia was performed via CO_2_ asphyxiation (30% chamber volume displacement rate) followed by cervical dislocation to ensure death. Tumor size and animal health were monitored daily by trained personnel. All experimental procedures were approved by the Animal Experiment Committee of Jing’an District Center Hospital of Shanghai.

### Statistical analysis

Statistical analysis was performed using SPSS software version 26.0 or GraphPad Prism 8. For normally distributed data, t-tests or Analysis of Variance (ANOVA) were used to test whether differences between groups were significant. Paired two-tailed Wilcoxon signed rank test was used for non-normally distributed data. Correlations were analyzed using the two-tailed Spearman parametric correlation test. A p-value of less than 0.05 was considered statistically significant.

## Results

### Up-regulation of lncRNA H19 expression is implicated in acquired drug resistance in glioblastoma multiforme

**Figure 1:**
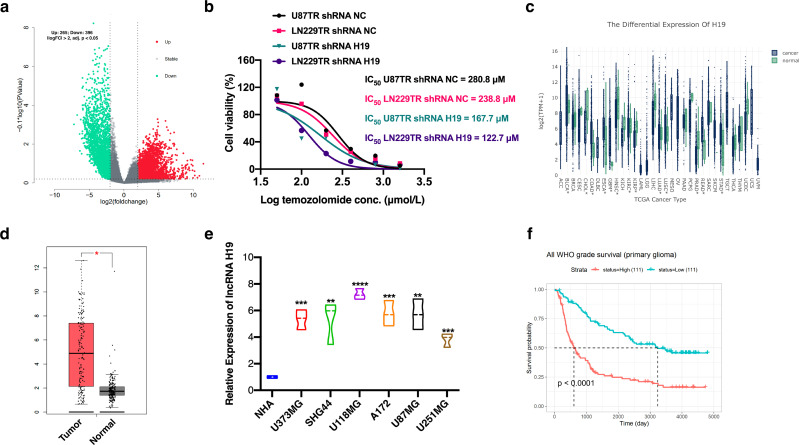
Up-regulation of lncRNA H19 expression correlates with acquired resistance to GBM. **(a)** differentially expressed lncRNAs in TMZ-resistant GBM cells. adj. P.Val < 0.01, log_2_FoldChange > 2 defines expression of up-regulated lncRNAs, adj. P.Val < 0.01, log_2_FoldChange < -2 defines expression of down-regulated lncRNAs. log_2_FoldChange < -2 defines the expression of down-regulated lncRNAs. Software: RStudio, 2022.07.1. The data comes from the GSE100736 dataset. **(b)** The IC50 values of U87TR and LN229TR cells for TMZ after knockdown of lncRNA H19 were determined using the CCK-8 assay. **(c)** lncRNA H19 expression levels in different tumor tissues and normal tissues were compared. *P<0.05. **(d)** Comparison of the expression levels of lncRNA H19 in GBM tumor tissues and non-tumor tissues. Control data were obtained from TCGA normal and GTEx data. **(E)** Comparison of the expression level of lncRNA H19 in different cells. normal human astrocyte (NHA). *P<0.05, **P<0.01, ***P<0.001, ****P<0.0001, compared with NHA group. **(f)** All WHO grade survival of primary glioma. Data from CGGA.

Analysis of the GSE100736 dataset revealed 265 up-regulated and 396 down-regulated lncRNAs associated with TMZ resistance development in GBM (**Figure 1A**). Among these, the top 20 significantly up-regulated lncRNAs included lncRNA H19, which exhibited the most pronounced increase (**Table 1**). To validate its expression in TMZ-resistant cells, we induced TMZ resistance in U87 and LN229 cells (U87TR and LN229TR) *in vitro*. QRT-PCR analysis confirmed significant up-regulation of lncRNA H19 (**Supplementary Figures 1A, B**). Knockdown of lncRNA H19 using shRNA in U87TR and LN229TR cells increased TMZ sensitivity (**Figure 1B**). High expression of lncRNA H19 was also observed in GBM tumor tissues according to TCGA-LIHC data (**Figures 1C, D**). *In vitro* studies demonstrated significantly higher lncRNA H19 expression in various GBM cell lines compared to normal human astrocytes (NHA) (**Figure 1E**). Moreover, elevated lncRNA H19 levels correlated with poor prognosis in primary GBM patients (**Figure 1F**). These findings underscore the association between increased lncRNA H19 expression and acquired TMZ resistance in GBM cell lines and tissues.

**Table 1:** Up-regulated expression of lncRNAs associated with acquisition of TMZ resistance in GBM (top 20 in descending order of logFC).

Gene_symbol	logFC	adj.P.Val
H19	14.20	4.68E-08
LINC00162	8.04	6.01E-07
RP3-523E19.2	7.54	4.60E-07
LINC00473	7.17	6.76E-08
RP11-119D9.1	6.62	6.13E-08
FAM201A	6.11	6.48E-06
RP11-49I11.1	6.09	1.54E-07
LINC00958	6.02	1.88E-07
RP11-706O15.5	5.95	2.20E-07
CH17-360D5.3	5.87	1.01E-07
LINC00987	5.82	3.42E-06
BASP1P1	5.76	1.57E-07
LINC01021	5.58	1.40E-07
CASC19	5.58	3.81E-06
RP11-115D19.1	5.49	7.58E-07
RP11-66B24.7	5.47	4.37E-07
RP11-90D4.3	5.44	2.80E-07
TINCR	5.41	8.60E-08
RP11-736N17.10	5.29	3.43E-06
AC006262.5	5.28	1.31E-06

### Knockdown of lncRNA H19 restores TMZ sensitivity in TMZ-resistant GBM cells *in vitro*

**Figure 2:**
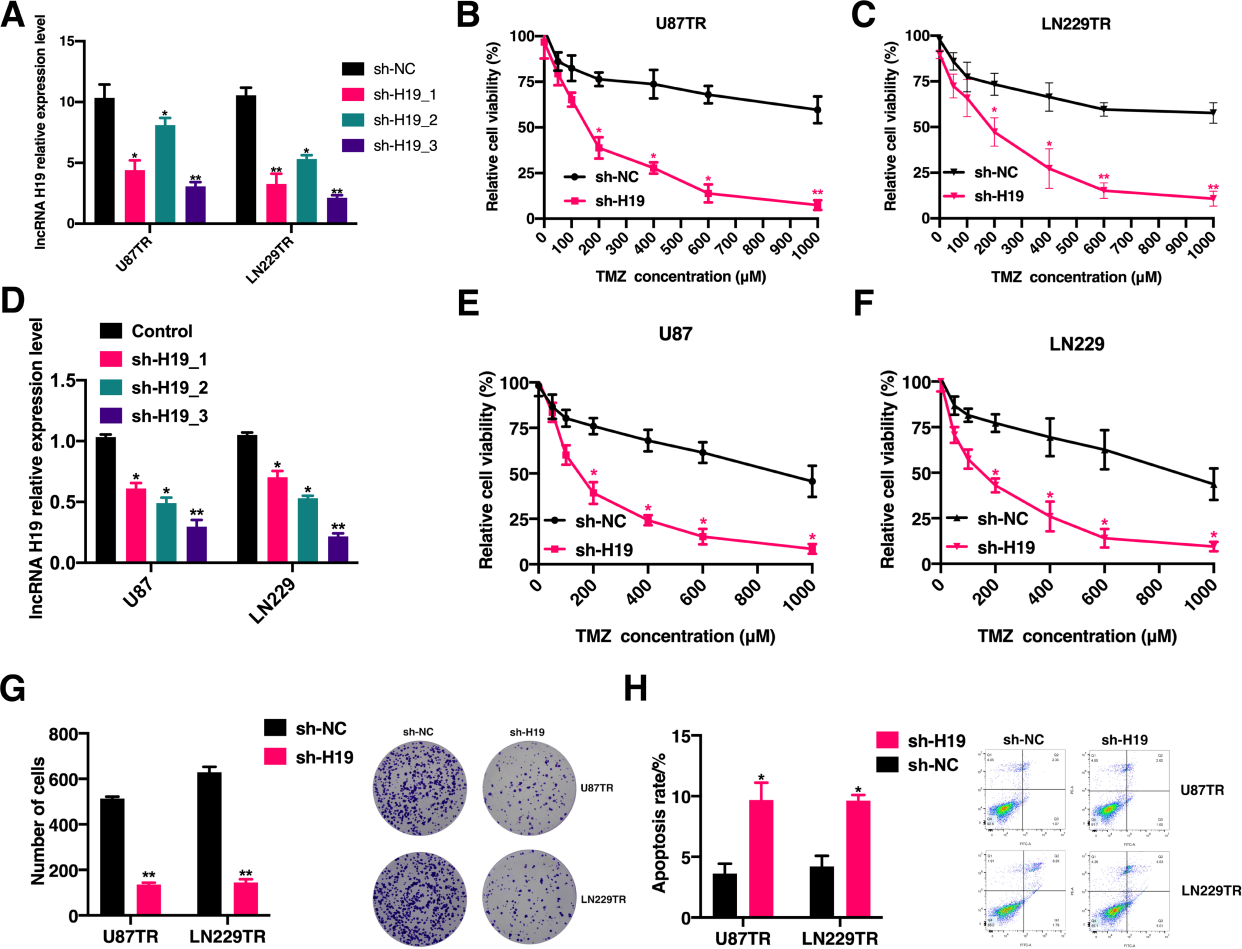
Knockdown of lncRNA H19 restores the sensitivity of TMZ-resistant GBM cells to TMZ *in vitro*. **(A)** qRT-PCR was performed to detect the relative expression levels of lncRNA H19 in U87TR and LN229TR cells after sh-H19_1, sh-H19_2, sh-H19_3 transfection. **(B)** CCK-8 assay to detect cell viability in U87TR cells after sh-H19 transfection. **(C)** CCK-8 assay to detect cell viability after transfection of sh-H19 in LN229TR cells. **(D)** qRT-PCR assay to detect the relative expression levels of lncRNA H19 in U87 and LN229 cells after sh-H19_1, sh-H19_2, and sh-H19_3 transfection. **(E)** Cell viability in U87 cells after transfection with sh-H19 was detected by CCK-8 assay. **(F)** Cell viability in LN229 cells after transfection with sh-H19 was detected by CCK-8 assay. **(G)** Clone formation rate of cells after sh-H19 transfection in U87 cells and LN229 cells after TMZ treatment for 3 weeks. **(H)** Comparison of apoptosis between U87TR cells and LN229TR cells after sh-H19 transfection in cells treated with TMZ. *p<0.05, **p<0.01, compared with the sh-NC group.

To elucidate the mechanism underlying acquired resistance to TMZ, we transduced three independent H19 shRNAs into U87TR and LN229TR cells. Following qRT-PCR analysis, sh-H19_3, which exhibited the highest inhibitory efficiency (**Figure 2A**), was selected for subsequent experiments. *In vitro* studies demonstrated a significant reduction in cell viability upon lncRNA H19 knockdown in U87TR and LN229TR cells (**Figures 2B, C**). Furthermore, TMZ stimulation following lncRNA H19 knockdown in U87 and LN229 cells (**Figure 2D**) markedly enhanced TMZ sensitivity and suppressed cell viability (**Figures 2E, F**). Colony formation assays revealed a substantial decrease in the clonogenic potential of U87TR and LN229TR cells following lncRNA H19 knockdown and TMZ treatment (**Figure 2G**). Moreover, the proportion of apoptotic cells was significantly higher in the knockdown group compared to the control group (**Figure 2H**). These findings indicate that knockdown of lncRNA H19 restores TMZ sensitivity in TMZ-resistant GBM cells *in vitro*.

### Overexpression of lncRNA H19 promotes acquisition of TMZ resistance in GBM cells

**Figure 3:**
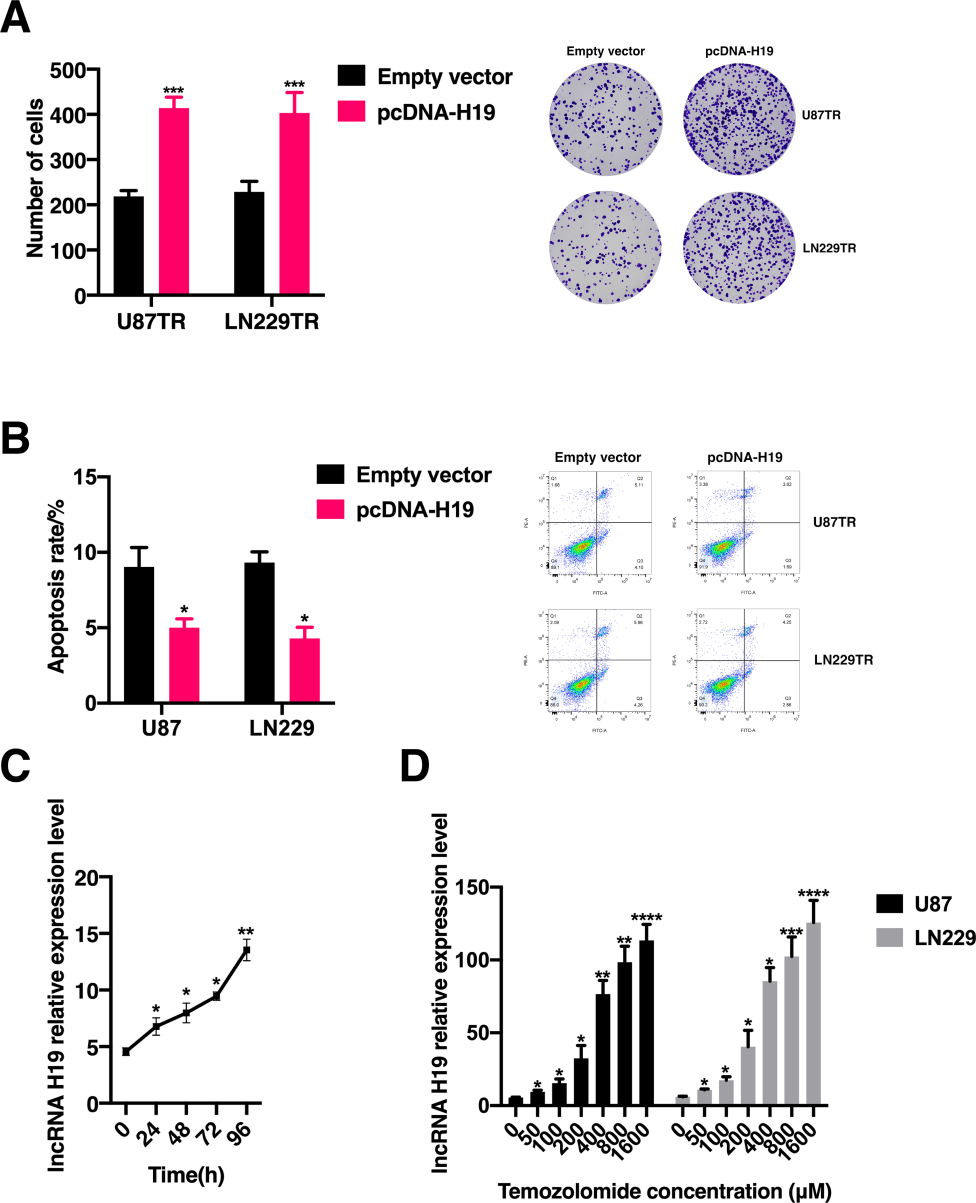
LncRNA H19 overexpression contributes to the acquisition of TMZ resistance in GBM cells. **(A)** Following treatment with 400 μM TMZ, the clone formation assay was conducted to assess cell proliferation in U87TR and LN229TR cells transfected with either the empty vector or a lncRNA H19 overexpression plasmid. Statistical significance (*p < 0.05) was observed compared to the Empty vector group. **(B)** After treatment with 400 μM TMZ, flow cytometry was employed to evaluate cell apoptosis in U87TR and LN229TR cells transfected with the empty vector or a lncRNA H19 overexpression plasmid. Statistical significance (*p < 0.05) was observed compared to the Empty vector group. **(C)** The relative expression levels of lncRNA H19 were measured in U87TR and LN229TR cells after 96 hours of treatment with 50 μM Temozolomide. Statistical significance was denoted as follows: *p < 0.05, **p < 0.01, ***p < 0.001, ****p < 0.0001, compared to the 0-hour group. **(D)** The relative expression levels of lncRNA H19 were measured in U87TR and LN229TR cells after 24 hours of treatment with varying concentrations of Temozolomide, Statistical significance was denoted as follows: *p < 0.05, **p < 0.01, ***p < 0.001, ****p < 0.0001, compared to the 0 μM Temozolomide treatment group.

To further investigate whether lncRNA H19 facilitates the acquisition of TMZ resistance in GBM cells, we generated lncRNA H19-overexpressing GBM cells (**Supplementary Figure 1C**). We assessed cell proliferation and apoptosis in U87TR and LN229TR cells following transfection with lncRNA H19. The results revealed a significant increase in the clonogenic potential of lncRNA H19-overexpressing U87TR and LN229TR cells (**Figure 3A**), accompanied by a notable decrease in apoptosis rates (**Figure 3B**). Moreover, short-term drug stimulation experiments demonstrated a time- and dose-dependent relationship between TMZ exposure and lncRNA H19 expression: longer TMZ exposure durations correlated with higher relative lncRNA H19 expression levels, and increasing TMZ doses corresponded to elevated lncRNA H19 expression levels (**Figures 3C, D**). These findings suggest that lncRNA H19 overexpression promotes GBM cell proliferation and suppresses TMZ-induced apoptosis, thereby contributing to the development of TMZ resistance in GBM cells.

### LncRNA H19 promotes TMZ resistance in GBM cells by sequestering hsa-miR-138-5p and hsa-miR-22-3p

**Figure 4:**
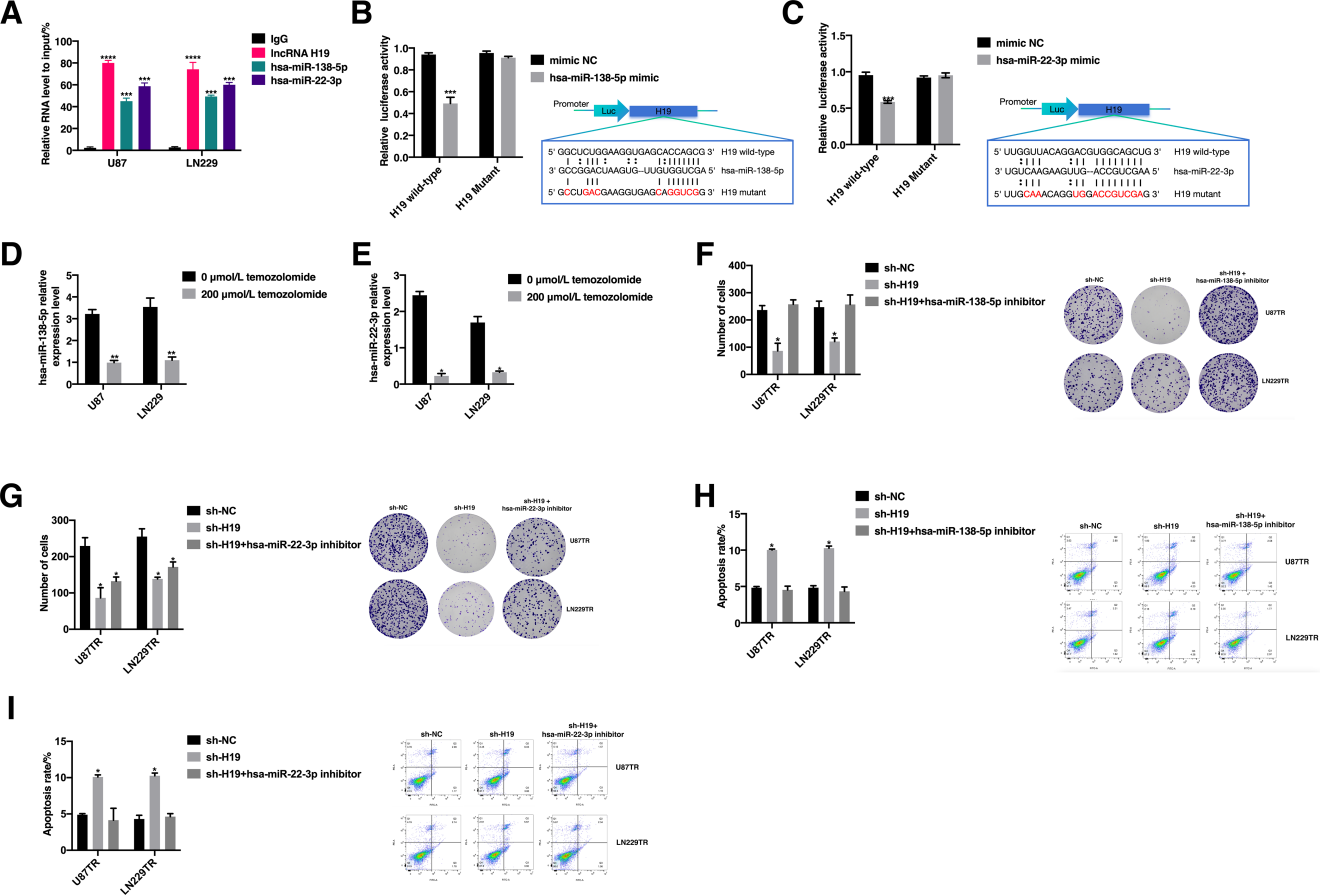
LncRNA H19 can promote the acquisition of TMZ resistance in GBM cells by “sponging” hsa-miR-138-5p and hsa-miR-22-3p. **(A)** RNA-binding protein immunoprecipitation (RIP) assay was performed to analyze the binding of lncRNA H19, hsa-miR-138-5p and hsa-miR-22-3p to Argonaut-2 (Ago2). ***p<0.001, compared with IgG group. **(B)** Results of luciferase reporter gene assay to detect the interaction of lncRNA H19 with hsa-miR-138-5p. ***p<0.001, compared with mimic NC. **(C)** Results of luciferase reporter gene assay to detect the interaction of lncRNA H19 with hsa-miR-22-3p, ***p<0.001, compared with mimic NC. **(D)** Comparison of hsa-miR-138-5p expression levels in U87 and LN229 cells after 200 μM temozolomide stimulation, **p<0.01, compared with 0μM temozolomide. **(E)** Comparison of hsa-miR-22-3p expression levels in U87 and LN229 cells after 200 μM temozolomide stimulation, **p<0.01, compared with 0μM temozolomide. **(F)** Comparison of clone formation rates in U87TR cells and LN229TR cells after co-transfection with sh-NC, sh-H19, and sh-H19+hsa-miR-138-5p inhibitor, *p<0.05, compared with sh-NC after treatment with 400 μM TMZ for 2 weeks. **(G)** Comparison of clone formation rates of U87TR cells and LN229TR cells after co-transfection with sh-NC, sh-H19, and sh-H19+hsa-miR-22-3p inhibitor after treatment with 400 μM TMZ for 2 weeks, *p<0.05, compared with sh-NC. **(H)** Comparison of apoptosis rates of U87TR cells and LN229TR cells after co-transfection with sh-NC, sh-H19 as well as sh-H19+hsa-miR-138-5p inhibitor after treatment with 400 μM TMZ for 24 hours, *p<0.05, compared with sh-NC. **(I)**. Comparison of apoptosis rates of U87TR cells and LN229TR cells after co-transfection with sh-NC, sh-H19 as well as sh-H19+hsa-miR-22-3p inhibitor after treatment with 400 μM TMZ for 24 hours, *p<0.05, compared with sh-NC.

Previous studies have established that lncRNA H19 acts as a competitive endogenous RNA, regulating downstream target gene expression to fulfill its biological roles (38, 39). RNA-binding protein immunoprecipitation (RIP) analysis demonstrated direct binding of lncRNA H19, hsa-miR-138-5p, and hsa-miR-22-3p to Argonaut-2 (Ago2) (**Figure 4A**). Prediction using the Encyclopedia of RNA Interactomes (ENCORI) and lncBase v.3 databases identified 29 miRNA binding sites on lncRNA H19. Analysis of data from the GSE100775 dataset revealed that downregulation of hsa-miR-138-5p and hsa-miR-22-3p correlated with TMZ resistance. Consequently, we focused on studying hsa-miR-138-5p and hsa-miR-22-3p further. Dual luciferase reporter assays confirmed the binding of hsa-miR-138-5p and hsa-miR-22-3p to predicted sites on lncRNA H19. Luciferase activity driven by lncRNA H19 was inhibited by hsa-miR-138-5p and hsa-miR-22-3p (**Figures 4B, C**). Moreover, expression levels of hsa-miR-138-5p and hsa-miR-22-3p were significantly reduced in U87 and LN229 cells following TMZ exposure (**Figures 4D, E**). To further investigate the role of hsa-miR-138-5p and hsa-miR-22-3p in lncRNA H19-mediated TMZ resistance in GBM cells, we overexpressed these miRNAs in lncRNA H19 knockdown U87TR and LN229TR cells (**Supplementary Figures 1D, E**). After treatment with 400 μM TMZ, the rate of clone formation in cells co-transfected with hsa-miR-138-5p inhibitor and sh-H19 showed no significant difference compared to controls (p > 0.05) (**Figure 4F**). Co-transfection with hsa-miR-22-3p inhibitor significantly enhanced clone formation compared to sh-H19 alone (p < 0.05), though it did not differ significantly from sh-NC (**Figure 4G**). After treatment with 400 μM TMZ, apoptosis assays showed significantly increased apoptosis rates in cells transfected with sh-H19 alone compared to controls (**Figure 4H**), and significantly decreased apoptosis rates in cells co-transfected with hsa-miR-22-3p inhibitor and sh-H19 (**Figure 4I**). These findings suggest that lncRNA H19 acts as a molecular sponge for hsa-miR-138-5p and hsa-miR-22-3p, and that these miRNAs can reverse the acquired TMZ resistance induced by lncRNA H19 in GBM cells.

### BMP2 is identified as a target gene of hsa-miR-138-5p, pivotal in the lncRNA H19-mediated pathway facilitating TMZ resistance in GBM cells

**Figure 5:**
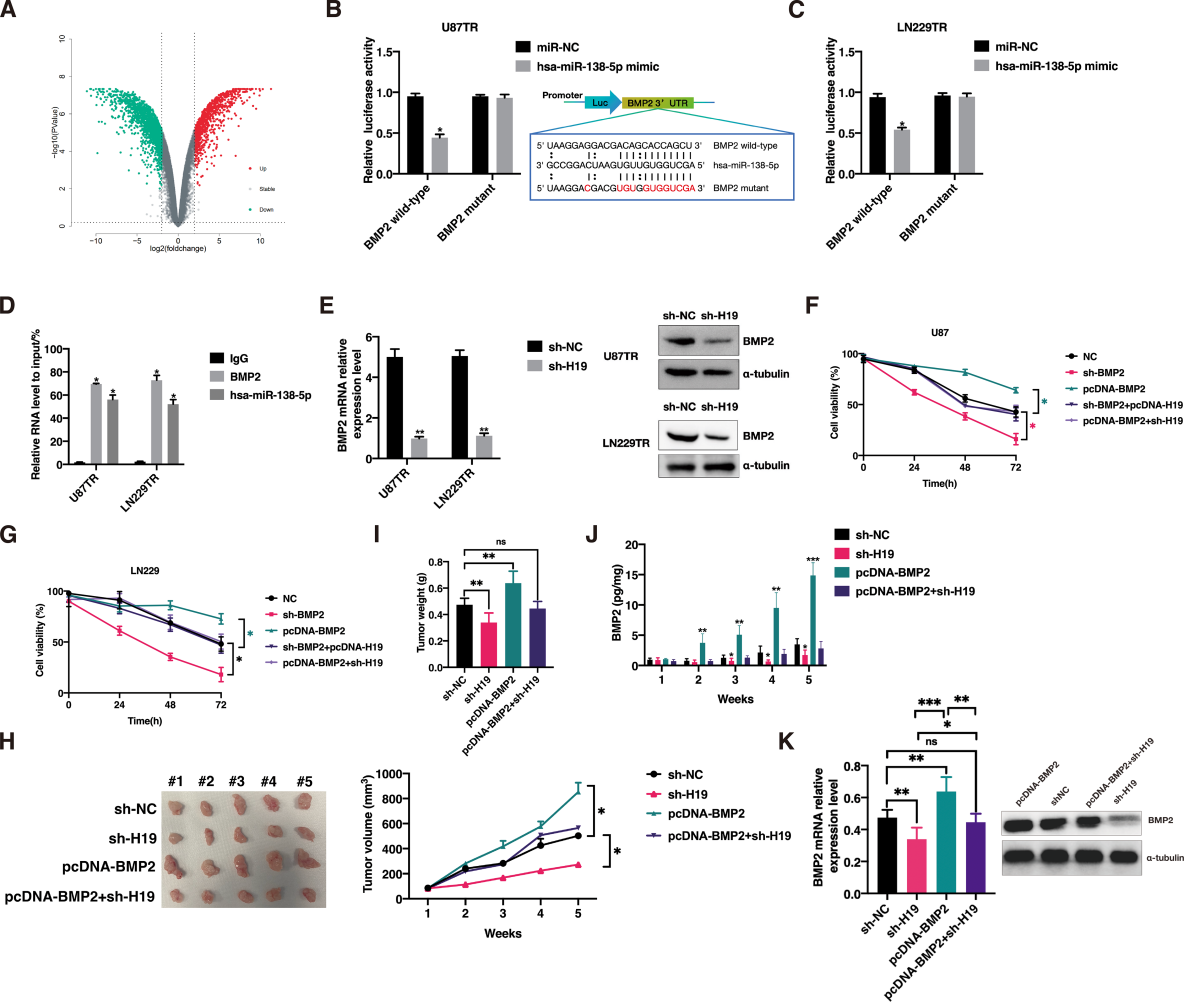
BMP2 is a key gene in the lncRNA H19-mediated pathway for GBM cells to acquire TMZ resistance. Knockdown of lncRNA H19 restores TMZ sensitivity *in vivo*, while BMP2 overexpression restores TMZ resistance. **(A)** GSE100736 dataset screened for volcano maps of genes associated with TMZ resistance. The luciferase reporter gene assay was used to detect the interaction between hsa-miR-138-5p and the BMP2 3′-UTR region in U87TR cells **(B)** and LN229TR cells **(C)**. **(D)** RNA-binding protein immunoprecipitation (RIP) assay to analyze the binding of BMP2 and hsa-miR-138-5p to Argonaut-2 (Ago2). **(E)** Expression of BMP2 was detected by qPCR and western Blot after transfection of sh-H19 in U87TR cells and LN229TR cells. **(F)** CCK-8 assay was used to detect the cell viability of U87 cells after co-transfection with no template control (NC), sh-BMP2, sh-BMP2+pcDNA-H19, and pcDNA-BMP2+sh-H19. *p<0.05. **(G)** CCK-8 assay was used to detect the cell viability of LN229 cells after co-transfection with NC, sh-BMP2, sh-BMP2+pcDNA-H19, and pcDNA-BMP2+sh-H19. *p<0.05. **(H)** Comparison of subcutaneous implant tumor size and volume in sh-NC, sh-H19, pcDNA-BMP2, pcDNA-BMP2+sh-H19 groups, n = 5. **(I)** Comparison of subcutaneous implant tumor mass in sh-NC, sh-H19, pcDNA-BMP2, pcDNA-BMP2+sh-H19 groups, n=5. **(J)** Comparison of serum BMP2 levels in mice in sh-NC, sh-H19, pcDNA-BMP2, pcDNA-BMP2+sh-H19 groups, n = 5. *p<0.05, **p<0.01, ***p<0.001, compared with sh-NC group. **(K)** Comparison of BMP2 levels in tumor tissues of mice in sh-NC, sh-H19, pcDNA-BMP2, pcDNA-BMP2+sh-H19 groups, n = 5. **p<0.01, ***p<0.001.

Initially, our focus was on identifying downstream targets of hsa-miR-138-5p. Using the GSE100736 dataset, we screened 867 genes associated with TMZ resistance (**Figure 5A**). Among the top 25 upregulated genes, BMP2 emerged as a potential target of hsa-miR-138-5p, based on predictions from ENCORI (40).

Subsequent qPCR assays confirmed that overexpression of hsa-miR-138-5p significantly suppressed BMP2 expression in U87TR and LN229TR cells, whereas BMP2 levels were notably elevated upon transfection with hsa-miR-138-5p inhibitor (**Supplementary Figure 1F**). Dual luciferase reporter assays demonstrated direct binding of hsa-miR-138-5p to the 3’-UTR region of BMP2 (**Figures 5B, C**). Moreover, RIP analysis revealed physical interaction between BMP2 and hsa-miR-138-5p bound to Ago2 (**Figure 5D**).

Additionally, knockdown of lncRNA H19 in U87TR and LN229TR cells resulted in significant BMP2 downregulation (**Figure 5E**). To assess BMP2’s role in the lncRNA H19-mediated TMZ resistance pathway, we manipulated BMP2 expression in U87 and LN229 cells (**Supplementary Figures 1G, H**). Results indicated that TMZ sensitivity increased significantly in cells transfected with sh-BMP2, whereas co-transfection with sh-BMP2 and pcDNA-H19 did not alter TMZ sensitivity compared to controls (p>0.05) (**Figures 5F, G**). These findings underscore BMP2’s critical involvement in the lncRNA H19-mediated pathway enabling TMZ resistance in GBM cells.

### Knockdown of lncRNA H19 restores TMZ sensitivity *in vivo*, whereas BMP2 overexpression restores TMZ resistance

To investigate the effects of lncRNA H19 knockdown and BMP2 overexpression on *in vivo* TMZ resistance, we conducted subcutaneous implantation experiments using stable transfected U87TR cells with sh-H19, sh-NC, pcDNA-BMP2, or sh-H19+pcDNA-BMP2 in nude mice. Two weeks after model establishment, mice received intraperitoneal injections of TMZ (5 μg/g) every 3 days. After continuous incubation for 5 weeks, we observed a significant reduction in tumor volume and mass in the sh-H19 group, whereas tumor volume was notably enlarged and mass increased significantly in the pcDNA-BMP2 group. In contrast, the sh-H19 + pcDNA-BMP2 group showed no significant abnormal increase in tumor volume or mass (**Figures 5H, I**). BMP2 levels in mouse serum were measured using ELISA, revealing significantly lower levels in the sh-H19 group compared to sh-NC, significantly higher levels in the pcDNA-BMP2 group, and no significant difference between the sh-H19+pcDNA-BMP2 group and sh-NC (**Figure 5J**). Furthermore, we extracted proteins from tumor tissues and performed Western blot analysis to examine the expression level of BMP2. The results showed that the BMP2 expression level in the tumor tissues of the sh-H19 group was significantly lower than that in the sh-NC group, while the BMP2 expression level in the tumor tissues of the pcDNA-BMP2 group was significantly higher than that in the sh-NC, sh-H19, and pcDNA-BMP2+sh-H19 groups (*P*<0.05). Additionally, there was no significant difference in BMP2 expression between the sh-NC and pcDNA-BMP2+sh-H19 groups(*P*>0.05). We extracted total RNA from mouse tumor tissues using TRIzol and analyzed the correlation between H19, hsa-miR-138-5p, hsa-miR-22-3p, and BMP2 mRNA levels using qPCR. The results are presented in **Supplementary Figures 1I–M**. Our findings showed that the level of H19 in tumor tissues was negatively correlated with hsa-miR-138-5p and hsa-miR-22-3p (r = -0.50, *P* = 0.03; r = -0.55, *P* = 0.01), while H19 was positively correlated with BMP2 mRNA expression (r = 0.98, *P* < 0.001). Both hsa-miR-138-5p and hsa-miR-22-3p were significantly negatively correlated with BMP2 mRNA (r = -0.44, *P* = 0.0499; r = -0.52, *P* = 0.02). These findings indicate that lncRNA H19 knockdown restores TMZ sensitivity *in vivo*, whereas BMP2 overexpression reinstates TMZ resistance.

## Discussion

Malignant GBM represents a highly aggressive tumor with substantial resistance to chemotherapy, posing significant challenges in treatment (41, 42). Clinical practice often combines radiotherapy, chemotherapy, and surgery to improve patient prognosis and reduce recurrence rates (43, 44). Temozolomide (TMZ) serves as the primary chemotherapeutic agent for GBM, but its efficacy is compromised by emerging drug resistance (45–47). Therefore, understanding the molecular mechanisms underlying TMZ resistance is critical for developing effective therapeutic strategies.

Extensive research indicates a pivotal role of lncRNAs in GBM progression and drug resistance (48, 49). This study explores the involvement of lncRNA H19 in TMZ chemoresistance in GBM cells. Our findings reveal significantly elevated expression of lncRNA H19 in TMZ-resistant compared to TMZ-sensitive GBM patients (50), a trend similarly observed in constructed TMZ-resistant GBM cell lines. The upregulation of lncRNA H19 in both GBM cell lines and tissues suggests its association with acquired TMZ resistance. Importantly, silencing lncRNA H19 restored TMZ sensitivity in resistant GBM cells *in vitro*. Conversely, lncRNA H19 overexpression promoted GBM cell proliferation and suppressed TMZ-induced apoptosis, thereby fostering TMZ resistance. These insights shed light on the mechanisms underlying TMZ resistance in GBM, implicating lncRNA H19 in chromatin modification and epigenetic gene regulation (51–53).

Our study elucidates the molecular mechanism of LncRNA H19 in TMZ-associated drug resistance, an area that has received limited attention. Our key finding demonstrates that LncRNA H19 is significantly upregulated in TMZ-resistant tumors compared to TMZ-sensitive tumors in GBM patients. Knockdown of LncRNA H19 using specific siRNA in TMZ-resistant U87 and U251 cell lines restored sensitivity to TMZ, highlighting LncRNA H19 as a significant factor in GBM cell acquisition of TMZ resistance (50). Furthermore, LncRNA H19 exerts an oncogenic role by modulating autophagy, presenting a potential therapeutic target in GBM treatment (54). Additionally, LncRNA H19 may contribute to GBM initiation and progression, offering potential insights for mitigating disease advancement (55).

Bone morphogenetic protein (BMP) is well-established for inducing astrocyte differentiation both *in vitro* and *in vivo*. BMP has been shown to enhance GSC sensitivity to TMZ treatment (17, 18). However, GSC have developed mechanisms to evade BMP-induced differentiation, necessitating investigation into the underlying molecular mechanisms within GSC to enhance BMP-induced differentiation therapy. Our study identifies BMP2 as a downstream target regulated by LncRNA H19. Upregulation of BMP2 expression via modulation by hsa-miR-138-5p and hsa-miR-22-3p confers TMZ resistance in GBM cell lines. Knockdown of LncRNA H19 restores TMZ sensitivity, an effect reversed by BMP2 overexpression. Our findings underscore the critical role of the LncRNA H19/hsa-miR-138-5p/hsa-miR-22-3p/BMP2 pathway in TMZ resistance acquisition in GBM cells, suggesting targeting this pathway as a promising strategy to address TMZ resistance in GBM. A BMP-mediated differentiation therapy strategy emerges as a promising approach for treating malignant GBM.

This study also has some shortcomings. The current subcutaneous xenograft model does not accurately replicate the intracranial microenvironment of glioblastoma (GBM). Therefore, an *in situ* intracranial implantation model should be developed by injecting U87TR/LN229TR cells into the mouse brain to verify the functional role of the H19/BMP2 axis and its potential therapeutic implications. In the CDX modeling study, a TMZ-only treatment group should be included to enable a comprehensive therapeutic comparison.

## Conclusion

In conclusion, our study provides compelling evidence of the pivotal role of lncRNA H19 in regulating TMZ resistance in GBM, which correlates with BMP2 overexpression during the development of TMZ resistance. These findings illuminate the intracellular mechanisms involved in BMP2-mediated chemosensitization to TMZ in GBM, offering potential avenues for targeted therapeutic strategies against TMZ resistance in GBM. Ultimately, our findings hold promise for enhancing the therapeutic efficacy of BMP2-induced sensitization to TMZ in GBM, offering hope for patients afflicted by this devastating disease.

## Data Availability

The original contributions presented in the study are included in the article/**Supplementary Material**. Further inquiries can be directed to the corresponding author/s.

## Supplementary material

Supplementary Figure 1
**(A)** Expression levels of lncRNA H19 after transfection with shRNA H19 in U87 cells and U87TR cells. **(B)** Expression levels of lncRNA H19 after transfection with shRNA H19 in LN229 cells and LN229TR cells. **p<0.01, ***p<0.001. **(C)** qPCR detection of lncRNA H19 expression levels in U87TR cells and LN229TR cells after transfection with empty vector and lncRNA H19 overexpression vector, ***p<0.001, compared with Empty vector. **(D)** the levels of hsa-miR-138-5p in the no template control (NC) group and hsa-miR-138-5p overexpression group were detected by RT-qPCR. **(E)** the levels of hsa-miR-22-3p in the NC group and hsa-miR-22-3p overexpression group were detected by RT-qPCR. ***p<0.001, ****p<0.0001, compared with NC group. **(F)** Relative expression levels of BMP2 mRNA in U87TR and LN229TR cells transfected with miR-NC, hsa-miR-138-5p mimic, hsa-miR-138-5p inhibitor. **(G)** Relative expression levels of BMP2 mRNA in U87TR and LN229TR cells transfected with miR-NC. *p<0.05, compared in miR-NC group. **(H)** Expression levels of BMP2 mRNA and BMP2 protein after transfection of no template control (NC), BMP2 short hairpin RNA (sh-BMP2), and BMP2 overexpression plasmid (pcDNA-BMP2) in U87 cells and LN229 cells. *p<0.05, ***p<0.001, compared with NC group. **(I)** Correlation between H19 and hsa-miR-138-5p levels in mouse tumor tissues. **(J)** Correlation between H19 and hsa-miR-22-3p levels in mouse tumor tissues. **(K)** Correlation between H19 and BMP2 mRNA levels in mouse tumor tissues. **(L)** Correlation between hsa-miR-138-5p and BMP2 mRNA levels in mouse tumor tissues. **(M)** Correlation between hsa-miR-22-3p and BMP2 mRNA levels in mouse tumor tissues.

Supplementary Table 1 Primer sequences of lncRNA H19 shRNA.
