# Supplementary figures and images for: Targeting the dual miRNA/BMP2 network: LncRNA H19-mediated temozolomide resistance unveils novel therapeutic strategies in glioblastoma
Source: Front Oncol. 2025 Apr 14;15:1577221. doi: 10.3389/fonc.2025.1577221 (PMC12034693; doi:10.3389/fonc.2025.1577221)

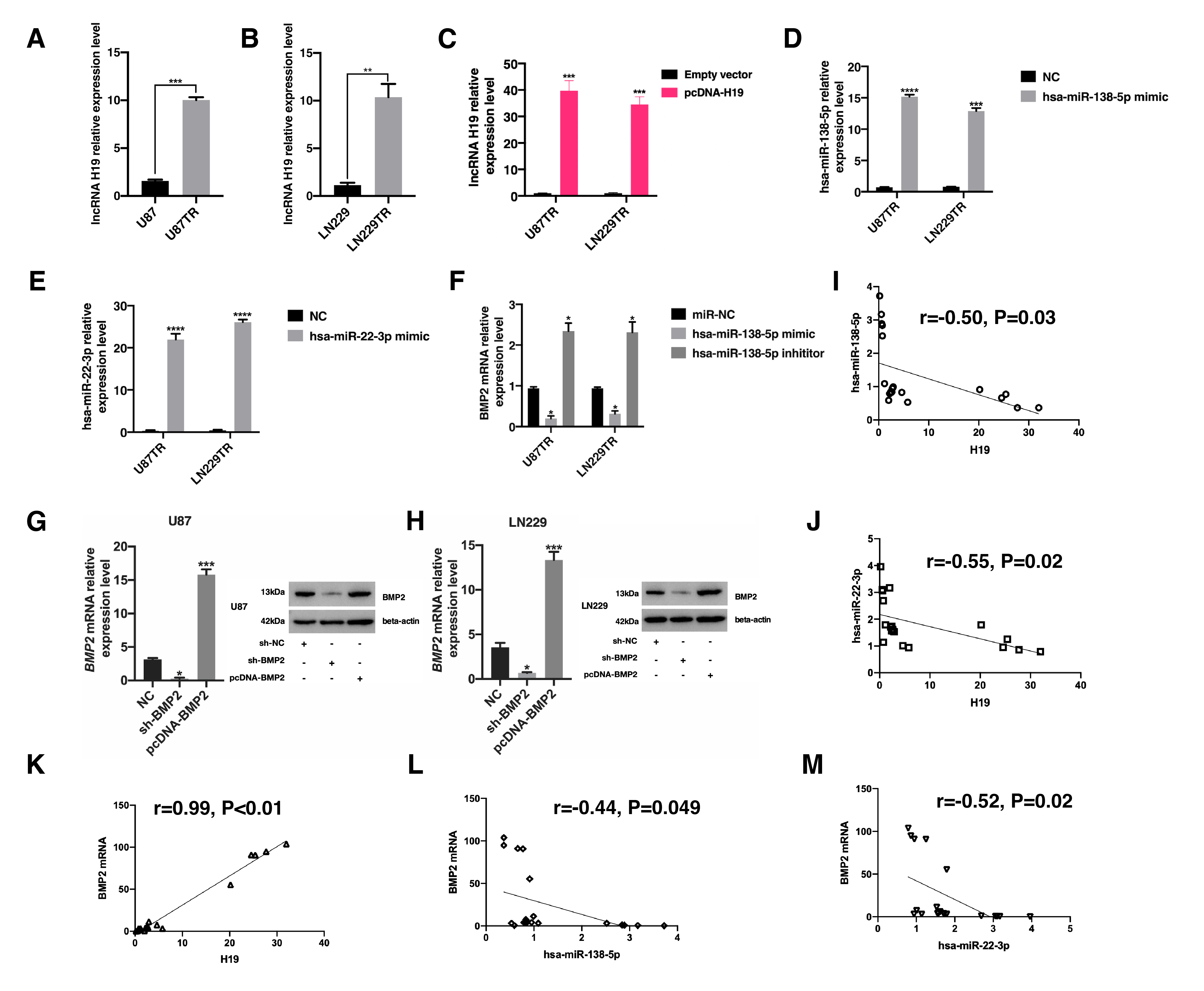

Supplement: Supplementary Figure 1 — (A) Expression levels of lncRNA H19 after transfection with shRNA H19 in U87 cells and U87TR cells. (B) Expression levels of lncRNA H19 after transfection with shRNA H19 in LN229 cells and LN229TR cells. **p<0.01, ***p<0.001. (C) qPCR detection of lncRNA H19 expression levels in U87TR cells and LN229TR cells after transfection with empty vector and lncRNA H19 overexpression vector, ***p<0.001, compared with Empty vector. (D) the levels of hsa-miR-138-5p in the no template control (NC) group and hsa-miR-138-5p overexpression group were detected by RT-qPCR. (E) the levels of hsa-miR-22-3p in the NC group and hsa-miR-22-3p overexpression group were detected by RT-qPCR. ***p<0.001, ****p<0.0001, compared with NC group. (F) Relative expression levels of BMP2 mRNA in U87TR and LN229TR cells transfected with miR-NC, hsa-miR-138-5p mimic, hsa-miR-138-5p inhibitor. (G) Relative expression levels of BMP2 mRNA in U87TR and LN229TR cells transfected with miR-NC. *p<0.05, compared in miR-NC group. (H) Expression levels of BMP2 mRNA and BMP2 protein after transfection of no template control (NC), BMP2 short hairpin RNA (sh-BMP2), and BMP2 overexpression plasmid (pcDNA-BMP2) in U87 cells and LN229 cells. *p<0.05, ***p<0.001, compared with NC group. (I) Correlation between H19 and hsa-miR-138-5p levels in mouse tumor tissues. (J) Correlation between H19 and hsa-miR-22-3p levels in mouse tumor tissues. (K) Correlation between H19 and BMP2 mRNA levels in mouse tumor tissues. (L) Correlation between hsa-miR-138-5p and BMP2 mRNA levels in mouse tumor tissues. (M) Correlation between hsa-miR-22-3p and BMP2 mRNA levels in mouse tumor tissues. [file Image1.tif]
