# Supplementary material for: Targeting the dual miRNA/BMP2 network: LncRNA H19-mediated temozolomide resistance unveils novel therapeutic strategies in glioblastoma
Source: Front Oncol. 2025 Apr 14;15:1577221. doi: 10.3389/fonc.2025.1577221 (PMC12034693; doi:10.3389/fonc.2025.1577221)
Supplement: Supplementary Table 1 — Primer sequences of lncRNA H19 shRNA. [file Table1.docx]

Supplementary Table 1 Primer sequences of lncRNA H19 shRNA

| siRNA_id | Forward Oligo Sequence | Reverse Oligo Sequence |
| --- | --- | --- |
| sh-H19_1 | CCGGCCCGTCCCTTCTGAATTTAATCTCGAGATTAAATTCAGAAGGGACGGGTTTTTG | AATTCAAAAAGCTGACAGAAAGAGCAATGATCTCGAGATTAAATTCAGAAGGGACGGG |
| sh-H19_2 | CCGGCCTGGACTCATCATCAATAAACTCGAGTTTATTGATGATGAGTCCAGGTTTTTG | AATTCAAAAACCTGGACTCATCATCAATAAACTCGAGTTTATTGATGATGAGTCCAGG |
| sh-H19_3 | CCGGCTCACCCACCGCAATTCATTTCTCGAGAAATGAATTGCGGTGGGTGAGTTTTTG | AATTCAAAAACTCACCCACCGCAATTCATTTCTCGAGAAATGAATTGCGGTGGGTGAG |
